# Design and rationale of the MR-INFORM study: stress perfusion cardiovascular magnetic resonance imaging to guide the management of patients with stable coronary artery disease

**DOI:** 10.1186/1532-429X-14-65

**Published:** 2012-09-19

**Authors:** Shazia T Hussain, Matthias Paul, Sven Plein, Gerry P McCann, Ajay M Shah, Michael S Marber, Amedeo Chiribiri, Geraint Morton, Simon Redwood, Philip MacCarthy, Andreas Schuster, Masaki Ishida, Mark A Westwood, Divaka Perera, Eike Nagel

**Affiliations:** 1 King’s College London, British Heart Foundation (BHF) Centre of Research Excellence, National Institute for Health Research (NIHR) Biomedical Research Centre at Guy’s and St. Thomas’ NHS Trust Foundation, Joint Imaging and Cardiovascular Divisions, Kings Health Partners, 4th Floor Lambeth Wing, St. Thomas' Hospital, London, SE1 7EH, UK; 2 Multidisciplinary Cardiovascular Research Centre & Leeds Institute of Genetics, Health and Therapeutics, University of Leeds, Leeds, United Kingdom; 3 NIHR Leicester Cardiovascular Biomedical Research Unit, Leicester, UK; 4 The London Chest Hospital, London, UK; 5 Department of Cardiology and Pneumology and Heart Research Center, Georg-August-University, Göttingen, Germany

**Keywords:** Perfusion, Cardiovascular magnetic resonance, Myocardial ischaemia, Fractional flow reserve, Stable angina

## Abstract

**Background:**

In patients with stable coronary artery disease (CAD), decisions regarding revascularisation are primarily driven by the severity and extent of coronary luminal stenoses as determined by invasive coronary angiography. More recently, revascularisation decisions based on invasive fractional flow reserve (FFR) have shown improved event free survival. Cardiovascular magnetic resonance (CMR) perfusion imaging has been shown to be non-inferior to nuclear perfusion imaging in a multi-centre setting and superior in a single centre trial. In addition, it is similar to invasively determined FFR and therefore has the potential to become the non-invasive test of choice to determine need for revascularisation.

**Trial design:**

The MR-INFORM study is a prospective, multi-centre, randomised controlled non-inferiority, outcome trial. The objective is to compare the efficacy of two investigative strategies for the management of patients with suspected CAD. Patients presenting with stable angina are randomised into two groups: 1) The FFR-INFORMED group has subsequent management decisions guided by coronary angiography and fractional flow reserve measurements. 2) The MR-INFORMED group has decisions guided by stress perfusion CMR. The primary end-point will be the occurrence of major adverse cardiac events (death, myocardial infarction and repeat revascularisation) at one year. Clinical trials.gov identifier NCT01236807.

**Conclusion:**

MR INFORM will assess whether an initial strategy of CMR perfusion is non-inferior to invasive angiography supplemented by FFR measurements to guide the management of patients with stable coronary artery disease. Non-inferiority of CMR perfusion imaging to the current invasive reference standard (FFR) would establish CMR perfusion imaging as an attractive non-invasive alternative to current diagnostic pathways.

## Background

Over the last few years, there have been a number of landmark trials questioning previously accepted management strategies for patients with stable coronary artery disease. The COURAGE [1] trial has shown no benefit of routine revascularisation both in terms of prognosis and long term symptom relief in patients with stable angina. However, subgroup analysis of the COURAGE data [2], suggests that the prognosis of patients receiving optimal medical therapy (OMT) alone or percutaneous coronary intervention (PCI) and OMT is correlated to their ischaemic burden and the amount of ischaemia reduction achieved by therapy, as assessed by single-photon emission computed tomography (SPECT). The assessment of prognosis, however, was exploratory, and not associated with outcome when adjusted for treatment.

There is evidence that the presence of stress-induced myocardial perfusion defects identified by non-invasive imaging tests such as SPECT have prognostic significance [3]. An observational study of 10 627 patients with stable angina and evidence of ischaemia as measured by SPECT described a survival advantage for revascularisation (of which 52% was PCI) over medical therapy in patients with ischaemia of more than 10% of the myocardium [4]. An alternative approach is the use of invasive fractional flow reserve (FFR) measurements to assess the functional significance of coronary stenoses. The FAME [5] and DEFER [6] trials have demonstrated that patients in whom angioplasty is only performed on FFR positive stenoses have an improved outcome compared to those patients in whom the revascularisation decision is based on a visual estimate of angiographic severity alone. The 2-year follow-up of the FAME patients showed a significant reduction in major cardiac events (MACE) in patients guided by FFR. Thus, there is accumulating evidence dictating a shift away from a purely anatomical assessment for coronary artery disease to a functional assessment. These data are of specific importance as most studies so far have determined diagnostic accuracy, whereas FAME and DEFER are among the few studies, where different diagnostic strategies were used to guide further management and thus improve outcome.

Cardiovascular magnetic resonance (CMR) perfusion is a well-established, non-invasive test with excellent accuracy for the detection of coronary artery stenoses [7] as well as abnormal FFR [8,9]. Multicentre data has demonstrated non-inferiority of CMR myocardial perfusion imaging in comparison to SPECT for the assessment of myocardial ischaemia [10]. In a recent single centre study of 750 patients the superiority of CMR perfusion in comparison to SPECT was shown mainly due to improved sensitivity (86.1% vs 65.5%) [11]. In addition, patients with a negative perfusion scan have a low likelihood (< 1%) of a major cardiac event over the following two years [12-14]. Thus, CMR perfusion seems well suited to guide the management of patients with stable coronary artery disease similar to invasive FFR measurements [15].

Although treatment decisions are increasingly being based on the combination of symptoms and objective proof of ischaemia, there is insufficient evidence on the best management strategy for patients with stable angina [16] and comparative effectiveness. The aim of the MR-INFORM study, therefore, is to establish whether guiding the management of patients with a moderate to high risk of coronary artery disease by CMR perfusion is non-inferior to guiding the management of these patients by invasive angiography and FFR.

## Methods

### Hypothesis

The principal hypothesis is that selecting patients with stable angina for revascularisation and optimal medical therapy (OMT) or OMT alone based on CMR myocardial perfusion is non-inferior to selecting patients based on routine coronary angiography and fractional flow reserve (FFR) in terms of subsequent major adverse cardiac events.

### Endpoints

#### Primary endpoint

The primary endpoint is the occurrence of a Major Adverse Cardiac Event (MACE) at one year. This is a composite end-point of death, myocardial infarction, and repeat target vessel revascularisation. See Table 1 for detailed definitions of endpoints.

**Table 1:** Detailed definition of end-points

**End Point**		**Definition**
Death		All cause mortality
Myocardial Infarction	Spontaneous	Elevation of CK or Troponin above baseline with symptoms of ischaemia, ECG changes or imaging evidence of loss of myocardium [22]
	Peri-procedural	CKMB > 3x ULN - upper limit of normal (post PCI 12-24 hrs) CKMB > 5x ULN (post CABG 24-72 hours) plus new Q waves or LBBB, new native vessel or graft occlusion, imaging evidence of loss of viable myocardium [22]
Repeat revascularisation		Repeat PCI or CABG of the target lesion performed for restenosis or other complication of the target lesion (from 5 mm proximal to 5 mm distal to the stent) [23]

### Secondary endpoints

• Symptoms (angina score, NYHA class) within 1 year.

• Death, myocardial infarction and repeat revascularisation as individual components of MACE.

• Cost-effectiveness of a CMR vs FFR guided selection for vascularisation.

• Occurrence of new myocardial scar tissue.

• Ischaemia reduction in the FFR vs CMR group after therapy.

### Study conduct and randomisation

MR-INFORM is a multi-centre, randomised controlled, non-inferiority trial. It is actively recruiting within the UK, Germany and Portugal. The study is performed in accordance with the principles of the Declaration of Helsinki; with all patients providing informed written consent prior to testing. The study protocol and other relevant documentation have been approved by the South London Ethics Committee (UK) and the relevant national ethics committees as well as registered on ClinicalTrials.gov identifier: NCT01236807: (http://clinicaltrials.gov/ct2/show/NCT01236807?term = MR + INFORM&rank = 1). Suitable patients are identified in the outpatient clinics and from the angiography and CMR waiting lists of the recruiting centres. If they meet the inclusion criteria and none of the exclusion criteria and provide written informed consent, they are enrolled into the study.

**Figure 1:**
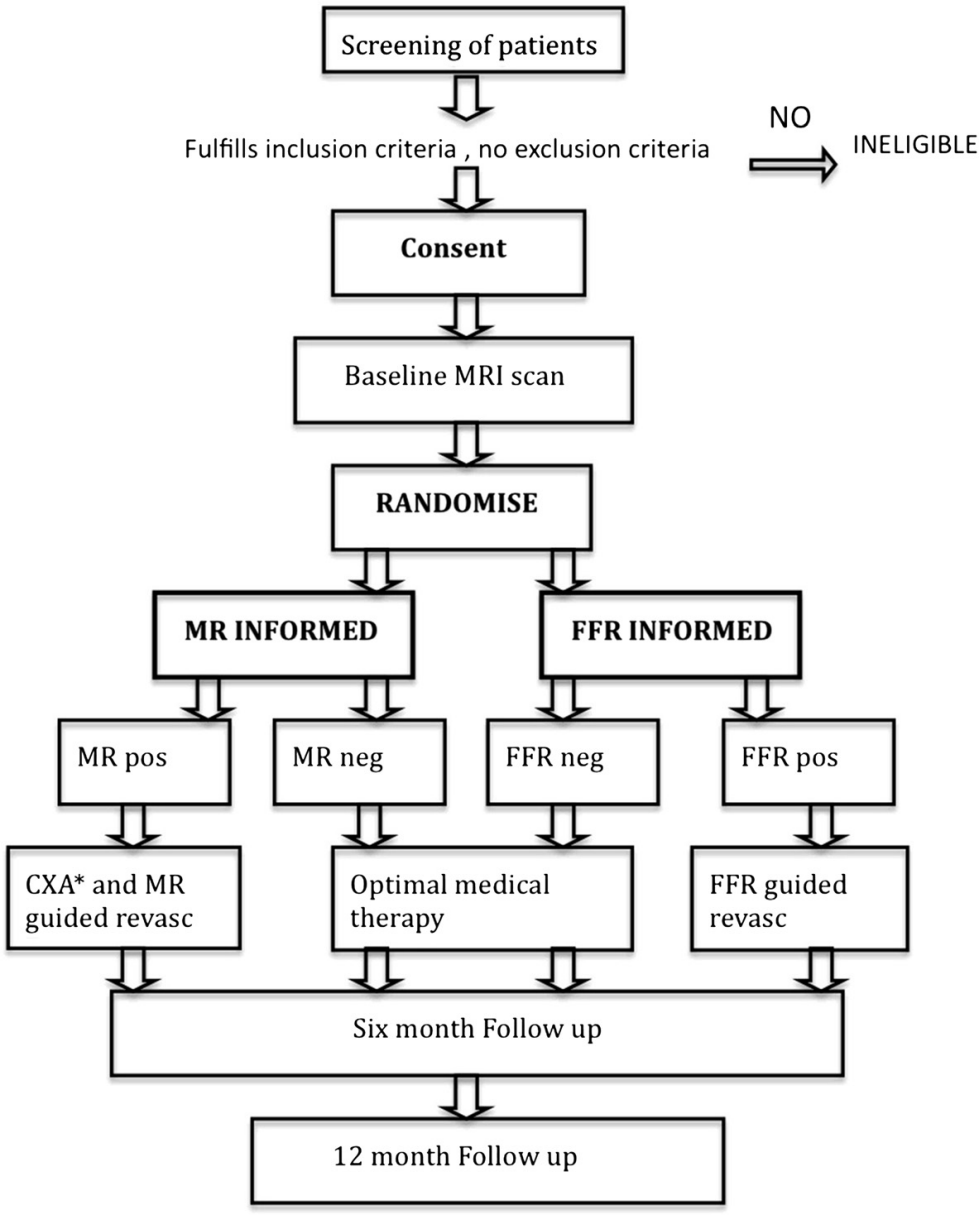
**Study Flow diagram outlining the study design.** *CXA = Coronary Angiography.

Patients are electronically randomised prior to the baseline CMR scan via a web-based randomisation service into randomly varying block sizes of 2 and 4, stratified by study site and gender with an allocation ratio 1:1 to MR-INFORMED or FFR- INFORMED (Figure 1).

### Patient population

Patients recruited into the study have typical symptoms of angina (Canadian Class Symptoms CCS 2-3), and either a positive exercise/bicycle test or more than two cardiovascular risk factors. Exclusion criteria include contra-indications to CMR imaging or to adenosine, poor renal function (eGFR < 30), LVEF < 30%, PCI within the last 6 months, and previous CABG. A prior myocardial infarction is not an exclusion criterion. The complete inclusion and exclusion criteria are listed in Table 2**.**

**Table 2:** Inclusion and Exclusion criteria

**Inclusion criteria**	**Exclusion criteria**
CCS Class 2 and 3*AND EITHER:* 2 or more of the following risk factors (diabetes, hypertension, smoking, family history, hypercholesterolaemia) *OR* Positive Exercise treadmill/bicycle test	Contra-indications to CMR
	Contra-indications to adenosine (AV-block2 or 3, symptomatic bradycardia, COPD with evidence of bronchospasm or asthma)
	Cardiac arrhythmias which may compromise image quality, atrial fibrillation or frequent ectopic beats > 20 bpm)
Age > 18 yrs	Known Left ventricular ejection fraction of less than 30%
Willing to undergo all study procedures	Persistent CCS class 4 angina
	NYHA class 3 and 4
	Previous coronary artery bypass grafts
	PCI within the previous 6 months
	Poor renal function (GFR < 30 ml/min) and /or allergy to contrast media
	Inability to lie supine for 60 mins
	Unstable medically
	Participating in any other clinical trial
	Pregnancy / breast feeding

All patients have a baseline stress perfusion CMR scan and further management will depend on whether they have been randomised to the MR-INFORMED or the FFR-INFORMED arm of the trial. In addition, all patients receive optimal medical therapy.

### FFR INFORMED group

Within the FFR-INFORMED group, the baseline CMR scan is blinded. This scan is not used to guide further management but will provide valuable additional scientific data that will be used in analyses specific to the substudies (described below). All patients in this group are examined with invasive angiography, FFR is performed in all arteries > 2.5 mm with a diameter stenosis > 40%. FFR is attempted in all arteries up to a 99% stenosis, as long as patency is not compromised. Although this strategy differs from clinical practice, which tends to focus more on intermediate lesions, evidence from the FAME trial suggests that even in severe and mild stenosis the visual estimate can be inaccurate [17]. The cut-off of 40% was used to ensure that all stenoses in the intermediate group i.e 50 -70% were interrogated with the pressure wire. The results of invasive angiography and the FFR are used to guide further treatment. Any narrowing with an FFR of > 0.8 is regarded as not haemodynamically significant, whereas a narrowing with an FFR of ≤ 0.8 or a total occlusion is regarded as significant and revascularisation is indicated. The decision on the mode of revascularisation is left to the treating Cardiologist and depends on local practice. In the case of repeat revascularisation, when symptoms are ongoing, revascularisation will be guided according to either the FFR or CMR results depending on the group randomised.

### MR- INFORMED group

Within the MR-INFORMED group, further management is dictated by the results of the baseline CMR scan. A visual assessment of the significance of ischaemia and the territories involved is made (see investigation reporting below). Patients with significant perfusion defects are referred for angiography and revascularisation is guided by the ischaemic territories identified on the CMR scan in combination with the results of the angiogram. FFR should not be done in this group.

### Baseline and follow-up visits

All patients undergo a thorough cardiovascular risk assessment at baseline with measurement of blood pressure, glucose levels, body mass index, including measurement of waist circumference, full lipid profile, the completion of a health questionnaire (EQ-5D) and a 12 lead ECG. Symptomatic status is recorded by CCS and NYHA class. Medical therapy is optimised in all patients and the risk factor assessments are repeated at 6 and 12 months to assess the impact of medical therapy.

Furthermore, a 6 month follow-up CMR scan will be done in a subgroup of patients to assess for residual ischaemia, new scar formation and changes in left ventricular function and size after the initial therapy recommended after the baseline investigations. The result of this CMR scan will remain blinded.

### Optimal medical therapy

At baseline, 6 months and 12 months recommendations for therapy are made to the primary care physician in line with guidelines published by the Joint British Societies [18]. See Table 3 for the treatment targets.

**Table 3:** Risk factor goals

**Risk Factor Goals**	
**Blood Pressure**	BP 130/80 mmHg
**Diabetes Mellitus**	Fasting or pre-prandial glucose of 4-6, or a HbA1C < 6.5%
**Lipids**	Total cholesterol less than 4 mmol/l or an LDL < 2.0 mmol/l
**Smoking**	Smoking cessation
**Weight**	BMI < 25

The goal of anti-hypertensive therapy is to achieve a blood pressure of less than 130/80 mmHg. The choice of anti-hypertensive therapy will be left to the treating physician.The aim of anti-lipid therapy is to achieve levels of LDL < 2 mmol/l and total cholesterol < 4 mmol/l. In the first instance, statin therapy will be initiated and then increased with the addition of a second agent if necessary. For patients with a BMI of > 25 kg/m^2^, the primary health care physicians are asked to refer the patient for dietary advice. Similarly, smokers are referred to the smoking cessation clinic. Recommendations for referral and for the continuation of therapy are made to the primary health care physician.

In patients without diabetes, who have a raised random glucose, the primary health care physician is asked to repeat a fasting glucose to assess for sustained hyperglycaemia, in which case the appropriate dietary advice and/or diabetic treatment should be instituted. In the case of diabetics with a raised blood sugar, the primary health care physician is asked to measure HbA1c and to ensure that the patients’ subsequent therapy is tailored to achieve a HbA1c of less than 6.5 mg/dl. All patients should be prescribed aspirin (clopidogrel if aspirin sensitive), statin therapy and an ACE Inhibitor [19].

### Investigations

#### CMR scanning

**Figure 2:**
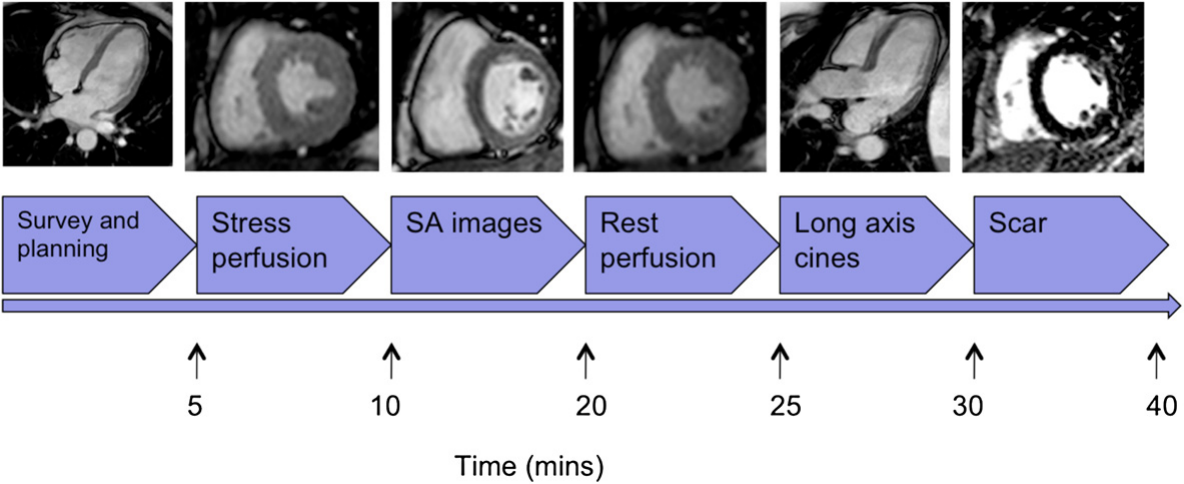
**MR-INFORM cardiac magnetic resonance protocol.** After individual patient planning using survey scans, intravenous adenosine is given according to the protocol. First pass stress perfusion imaging is done during stress visualizing the first passage of a 0.75 mmol/kg contrast agent bolus through the myocardium. Short axis (SA) cine images are acquired. Rest perfusion images are acquired during an injection of a second bolus of 0.75 mmol/kg contrast agent. 10 minutes after an additional injection of 0.05 mmol/kg of contrast agent to increase the total dose of contrast agent to 0.2 mmol/kg is given. A modified Look-Locker inversion time scout is performed prior to late gadolinium enhancement imaging in short axis and long axis views. During the wait time after the last contrast agent injection long axis images in the 4 chamber, 3 chamber and the 2 chamber views are obtained.

CMR imaging will be performed on 1.5 T scanners (various vendors); total scan duration will be approximately one hour. All images will be acquired using phased array surface coils during mild expiration and electrocardiographic triggering. Cine images will be acquired in the 4-, 3-, 2-chamber and contiguous short axis views using a steady state free precession (SSFP) sequence (Figure 2). For perfusion, a basal, midventricular, and apical short axis slice will be acquired during the first pass of 0.075 ml/kg of a 1-molar gadolinium based contrast agent injected with a power injector at a flow rate of 4mls/s. The exact details of the sequence will vary between different scanners used but will fulfill certain basic criteria, including high spatial resolution(< 3 mm x 3 mm), acquisition duration < 180 ms per slice, 90° saturation pre-pulse, fixed pre-pulse delay and dual bolus contrast injection allowing semi-quantitative and fully quantitative analysis [20]. Hyperaemia will be achieved by infusion of adenosine at 140mcg/kg/min for 3-4 minutes. If there is neither a heart rate increase > 10 bpm nor a systolic blood pressure drop > 10 mmHg from baseline nor symptoms the adenosine dose will be increased to 170 mcg/kg/min after 2 minutes and to a maximum dose of 210 mcg/kg/min [21]. All patients will be asked to avoid caffeine, but to continue with their normal medication for 12 hours prior to the scan. For the perfusion measurements, contrast agent will be injected at the third minute of adequate adenosine stress using a dual bolus method. A dose of 0.075 mmol/kg of a 1-molar gadolinium-chelate (Gadobutrol, Gadovist®, Bayer, Germany) will be injected for the main bolus preceded by the same volume of a 10% diluted contrast agent dose for a prebolus both flushed with 25 ml of saline. Rest perfusion imaging will be performed 10 min after stress perfusion to allow for clearance of most of the contrast agent.

Late enhancement imaging will then be carried out using an inversion recovery turbo gradient echo sequence.

### Coronary angiography and pressure wire measurements

At angiography, any arteries with a diameter of > 2.5 mm and a diameter stenosis between 40–99% will be assessed with FFR. A 0.014 guidewire (Radiwire, St. Jude Medical, St. Paul, MN, USA or Volcano, Volcano Corporation, San Diego, CA) will be introduced, calibrated and advanced into the coronary artery distal to the stenosis. An intravenous adenosine infusion (140 mcg/kg/min) will be used to induce maximal hyperaemia. FFR is calculated as the ratio of the mean distal coronary pressure measured by the guidewire to mean aortic pressure measured by the guiding catheter. Revascularisation will be recommended in the FFR group if FFR is < 0.80. In the case of a chronic total occlusion, FFR is regarded as positive and a default value of 0.5 is assigned to the chronically occluded vessel. In the case of triple vessel disease, an attempt should be made to do FFR in all three arteries.

### Investigation reporting

#### CMR analysis

The CMR analysis for the MR-INFORMED group will be done by the local supervising CMR physicianas per normal clinical practice.

The following data will be collected:

1) Image quality (assessed on a grade of 1 to 4 – poor to excellent).

2) Presence of scar tissue and transmurality (1 = 1-25%; 2 = 25-50%; 3 = 51-75%; 4 = > 75%) based on the AHA/ACC 17 segment model.

3) Perfusion analysis at stress and rest scored as significant, insignificant or no defect based on the AHA/ACC 16 segment model (see below).

4) Evidence of regional wall motion abnormalities (1 = akinetic; 2 = hypokinetic; 3 = dyskinetic).

5) Quantitative analysis including end-diastolic and end-systolic volumes and ejection fraction.

The baseline CMR scan in the FFR-INFORMED group will remain blinded.

### Perfusion analysis

The CMR perfusion images will be interpreted visually and angiography recommended if the perfusion defect is classified as significant according to the presence of ischaemia in 2 segments of a 32 segment model (see below) i.e:

• > 60 degrees in either the basal or the midventricular slices or

• > 90 degrees in the apical slice or

• any transmural defect or

• two adjacent slices.

In the case of patients who have unexpected scar tissue evident on CMR, angiography would be recommended for all patients who have evidence of scar and peri-infarct ischaemia. When only scar is present, the management will be decided after an assessment of the transmurality of scar. In patients with transmural scar (> 75%), and no additional ischaemia, angiography would not be recommended. In patients with partial transmurality (< 75%), and no ischaemia, the need for angiography will be decided by the treating physician after an assessment of the patient’s symptoms and risk. It is recommended, however, that the subsequent revascularisation strategy be guided by the areas of ischaemia alone.

The rationale for the threshold at which ischaemia is deemed to be significant is based on data from Hachamovitch et al. [4] who demonstrated that in patients with 10 – 12.5% ischaemic myocardium on SPECT, mortality is significantly higher if they are not revascularised in comparison to a revascularised group. The opposite is true in patients with less than 10% ischaemic myocardium.

With the high spatial resolution of CMR perfusion (< 3 mm x 3 mm) it is possible to distinguish endocardial, non-transmural, perfusion defects from transmural (> 75% wall thickness) perfusion defects. Thus, the myocardium can be divided into the 16 AHA/ACC segments with a subdivision into an endocardial and an epicardial half resulting in a total of 32 segments. Each of these segments represents approximately 3% of the myocardium, two positive segments represent approximately 6%. Since there is no data available providing a direct comparison between the ischaemic burden in SPECT and CMR perfusion this level is used as a conservative threshold to refer patients to invasive angiography.

The criteria for interpretation of CMR scans, especially the differentiation between normal myocardium, non-transmural defects, and transmural defects, will be discussed in detail with each site during the initiation visit using a set of example cases. In addition, CMR images will be periodically reviewed by the global coordinating investigator and selected coordinating investigators from participating countries who – in case of inconsistencies or differences in assessment – will contact the sites and consider specific training activities. Each site can invite a second opinion from the global coordinating investigator or a coordinating investigator from a participating country (or one of their experienced co-workers) in unclear cases.

### Invasive angiography

The results of the coronary angiogram will be assessed by the operator at the time of the angiogram. The following data will be collected:

• Coronary artery dominance

• Location and presence of stenosis by visual estimate

• Vessel diameter

• FFR measurement in all arteries > 2.5 mm with a diameter stenosis greater than 40% but not chronically occludedon visual assessment.

### Revascularisation

Revascularisation by either (PCI) or coronary artery bypass grafting (CABG) will be guided by the ischaemic territories identified by either FFR or CMR perfusion, depending on the randomisation group. For example, if the CMR perfusion scan demonstrates a perfusion defect in the left anterior descending (LAD) territory and angiographically a narrowing is identified in the LAD and right coronary artery (RCA), only the LAD artery will be revascularised.

In visual triple vessel disease, the FFR or CMR results will be used to guide the decision towards CABG or PCI.

If there is more than one artery that needs to be revascularised, the PCI procedure can be staged. If this occurs, follow-up will begin after the first procedure, and the endpoints will include both procedures.

Post-interventional FFR is useful but not mandated in order to keep the procedure as simple as possible.

The specific revascularisation technique, i.e. type of stents used, staging of the procedure, PCI or CABG etc. will be decided by the treating interventionalist and dictated by local guidelines. This is to reflect “real –world” practice and to acknowledge that practice varies between centres and countries. Similarly, the cardiac surgeons will decide on the type of surgical revascularisation that they will undertake.

In the case of patients who demonstrate scar and ischaemia on CMR perfusion subsequent revascularisation will be guided by areas of ischaemia alone rather than on the basis of scar.

### Substudy analyses

A number of substudies will be performed based on the data acquired during the main study. These will not interfere with the aims and the conduct of the main trial but will provide valuable additional data. The major substudies are:

1. Determination of an ischaemic threshold for revascularisation with semi-quantitative and fully quantitative perfusion analysis.

2. Correlation between the results of FFR and CMR perfusion.

3. Ischaemia reduction in the MR-INFORMED vs the FFR-INFORMED group

4. The effect of new scar formation on outcome.

5. The cost-effectiveness of an MR-INFORMED vs a FFR-INFORMED revascularisation strategy.

### Data collection and monitoring

Patient demographic details, medical history and information on current medication use will be collected. A 12 lead ECG will be performed at baseline and at 12 months. Blood tests will include a measurement of total cholesterol and full lipid profile, a random glucose measurement, and renal function (eGFR). These tests will be repeated at 6 and 12 months. A full blood count will also be measured at baseline. Blood pressure, waist circumference measurement, and Body Mass Index will be determined at baseline, 6 and 12 months.

At the time of revascularisation (PCI or CABG), baseline troponin will be measured. This will then be repeated 6 hours post PCI or 12 hours post CABG. If the troponin is raised, then a CKMB will be done at 12-24 hours post PCI or 36-72 hours post CABG. The definition of myocardial infarction is based on CKMB. See Table 1 for detailed definitions of end-points.

All study data is recorded via an electronic case report form (eCRF). Data will be monitored at all sites for completeness and quality by the contract research organization (CRO). A full data-monitoring schedule will be established and an independent data monitor will verify the eCRF against the source data.

Any adverse events or serious adverse events are recorded on the eCRF and forwarded to the sponsor and the CRO immediately. An independent Data Monitoring Committee will review serious adverse events and any other trial safety issues.

### Organisation

The trial has been approved by the ethics committees either nationally or locally depending on site stipulations in each country. A CRO has been contracted to oversee the monitoring of all sites, establishing the eCRF and checking the completeness and consistency of the trial data.

A trial steering group has been appointed to help the chief investigator with the scientific aspects of the trial and to oversee the progress of the trial. They will also advise on any major protocol modifications suggested by the investigators.

### Statistical considerations

#### Sample size

The sample size calculation is based on the primary endpoint of death, MI, and repeat revascularisation at one year. A 10% event rate in the FFR group and an equivalence margin of 6% were assumed based on the results of the FAME study [5]. A sample size of 826 would be required to determine the non-inferiority of aCMR guided strategy compared to an FFR guided strategy with at least 80% power. Allowing for a dropout rate of 10% a total sample size of 918 patients is necessary. The calculation was carried out using STATA 11SE.

We do anticipate that there will be crossover between groups, most likely from the CMR group to the FFR group, when patients continue to experience symptoms and the CMR scan has been reported as negative. This will be taken into consideration during the statistical analysis which will be done on a per protocol and an intention to treat basis.

### Data analysis

#### Primary analysis

The main objective of this study is to assess whether an “MR-INFORMED” management strategy is non-inferior to an “FFR INFORMED” strategy for the clinical management of patients with angina who are at moderate to high risk of CAD. To satisfy this objective the study will test the following null-hypothesis:

H_0_: The difference in MACE incidence rates between MRI and FFR group is above or equal to δ .

The outcome will be primarily assessed on an intention to treat basis (mITT) and secondarily on a per protocol (PP) analysis. The null hypothesis H_0_ can be rejected and non-inferiority of the MR-guided strategy claimed, if the two-sided 95% confidence interval (CI) for the difference in incidence rates is completely below δ in both the (mITT) and (PP) analysis. A difference of 6%-points was regarded as clinically relevant. Therefore the non-inferiority margin was set to 6%-points.

In addition, absolute and relative frequencies will be given per group. All statistical analyses will be carried out by an independent statistician. A formal interim analysis is not planned in this study.

For all secondary efficacy variables, descriptive statistics (n, mean, standard deviation, median, minimum, and maximum) will be calculated for each quantitative variable. Absolute and relative frequencies will be given for categorical data.

### Cost-utility analysis

The economic analysis will compare the total costs and effectiveness within each arm of the treatment and provide incremental cost effectiveness ratio (ICER). It will be computed as incremental costs divided by increment quality adjusted life years (QALY). The total costs will include both the direct and indirect costs. Costs will be computed by recording patient utilisation of all NHS services for the follow-up period of one year. These include, for instance, the costs of the initial diagnostic procedures (CMR, coronary angiography and FFR), revascularisation (PCI or CABG), hospitalisation costs, pharmacy costs, optimal medical therapy costs over the two years plus costs associated with any re-hospitalisation due cardiac events (MI, repeat revascularisation). These costs will be assessed using the NHS reference costs. Additional costs will be computed using patient diaries and will include direct health care expenditures plus total time off work (to compute lost income), travel time and any travel or work time costs incurred by home care givers (e.g. spouse, parent, etc.). Quality of Life assessment will be carried out using the EQ5D questionnaire and will be administered at base line and every two months for up to 24 months. Bootstrap methods will be used to derive confidence intervals around the ICER and to derive the Cost Effectiveness Acceptability Curve (CEAC).

### Study limitations

In this prospective outcome trial we address an important clinical question. There is currently insufficient evidence on the comparative effectiveness of different diagnostic strategies in patients with stable coronary artery disease and therefore we believe that the results of this trial will help to inform future guidelines.

In order to achieve this objective, the trial has been designed to reflect “real-world“ medical practice as closely as possible. This is important for the translation of its results into routine clinical practice. However, inevitably this leads to some limitations in the study design.

Although we aim at optimising medical therapy in both groups, there are variations on what is deemed optimal in different health systems and the implementation of this is left to the primary care physician. We follow the UK guidelines to minimise variation as much as possible, the remaining variation reflects clinical practice.

A critical point is the inclusion of peri-procedural myocardial infarction and target lesion revascularisation in our end-point. These endpoints are frequently used in interventional trials. The occurrence of peri-procedural myocardial infarctions seems an important end-point as they are associated with adverse outcome and may reflect better guidance by one technique or the other. However, as we guide revascularisation by objective proof of ischaemia in both arms, we expect that all patients in the trial are guided towards fewer revascularisation procedures compared to angiography alone and that this will be reflected in low event rates in both arms. The rationale for using target lesion revascularisation is similar.

It is difficult to predict the frequency of crossover from one arm to another, which can be substantial (e.g. COURAGE trial). We do not anticipate a high rate of cross-over, however, the data monitoring committee will assess the occurrence of cross-over and allow for adaptation of the randomisation scheme to account for this.

### Summary

MR–INFORM is an international, prospective, randomised controlled, non-inferiority outcome trial comparing the role of CMR perfusion to routine coronary angiography with invasive fractional flow measurements for guiding patients with stable angina and an intermediate to high likelihood of coronary artery disease. Non-inferiority of CMR perfusion imaging to the current invasive reference standard (FFR) would establish CMR perfusion imaging as an attractive, non-invasive alternative to current diagnostic pathways. The results will help to inform national and international guidelines on the investigation and management of coronary artery disease, and ultimately lead to improved patient care.

### Trial status

MR-INFORM is currently recruiting in the UK, Germany and Portugal. There are currently six sites active in the UK and one in Portugal and Germany. Another six German sites and three UK sites are currently undergoing ethics approval or contract negotiation and should be initiated soon. The number of patients recruited at the time of submission is 235.

## Abbreviations

AHA: American Heart Association; ACC: American College of Cardiology; ACE: Angiotensin Converting Enzyme inhibitor; AV: Atrio-Ventricular; BMI: Body Mass Index; CAD: Coronary Artery Disease; CABG: Coronary Artery Bypass Graft; CCS: Canadian Class Score; CEAC: Cost Effectiveness Acceptability Curve; CK: Creatinine Phosphokinase; CKMB: Creatinine Phosphokinase MB Isoenzyme; CMR: Cardiovascular Magnetic Resonance; COPD: Chronic Obstructive Pulmonary Disease; CRO: Contract Research Organisation; CTO: Chronic Total Occlusion; CXA: Coronary Xray Angiography; ECG: Electrocardiogram; eCRF: Electronic Case Report Form; eGFR: Estimated Glomerular Filtration Rate; FFR: Fractional Flow Reserve; ICER: Incremental Cost Effectiveness Ratio; ITT: Intention To Treat; LAD: Left anterior descending artery; LBBB: Left Bundle Branch Block; LDL: Low-Density Lipoprotein; RCA: Right Coronary Artery; MI: Myocardial Infarction; MACE: Major Adverse Cardiac Event; NYHA: New York Health Association; OMT: Optimal Medical Therapy; PCI: Percutaneous Coronary Intervention; PP: Per Protocol; QALY: Quality Adjusted Life Years; SA: Short Axis; SSFP: Steady State Free Precession; SPECT: Single-Photon Emission Computed Tomography; ULN: Upper Limit of Normal.

## Competing interests

Eike Nagel has received grant support from Philips Healthcare and Bayer Healthcare.

## Authors' contributions

SH, MP, EN have made substantial contributions to the conception and design of the study and helped draft the manuscript. AMS, MM, SP, AC, GM, ES, MW, DP, AS, MI participated in the design of the study. All authors read and approved the final manuscript.
